# Biliary stents can modify the microbiota and promote the progression of pancreatic cancer

**DOI:** 10.3389/fonc.2026.1633611

**Published:** 2026-01-29

**Authors:** Michele Fiordaliso, Barbara Pala, Giuseppe Marincola, Mariagrazia Piscione, Luca Savino, Mariangela Mazzone, Maria Carmela Di Marcantonio, Gabriella Mincione

**Affiliations:** 1 Department of Medicine and Ageing Sciences, University “G. d’Annunzio” of Chieti–Pescara, Chieti, Italy; 2 Division of Cardiology, Department of Clinical and Molecular Medicine, Sant’Andrea Hospital, Sapienza University of Rome, Rome, Italy; 3 Ph.D. School of Applied Medical-Surgical Sciences, University of Tor Vergata, Rome, Italy; 4 Division of General Surgery, San Camillo Forlanini Hospital, Rome, Italy; 5 Bio-Medico University of Rome, Rome, Italy; 6 Department of Biomedicine and Prevention, Institute of Anatomic Pathology, University of Roma Tor Vergata, Rome, Italy; 7 Department of Innovative Technologies in Medicine & Dentistry, University “G. d’Annunzio” of Chieti-Pescara, Chieti, Italy

**Keywords:** biliary stent and pancreas microbioma, chemotherapy resistance, dysbiosis and pancreatic cancer, gut microbiota, microbiota modulation, pancreatic cancer

## Abstract

Pancreatic cancer (PC), the fourth cause of cancer-related deaths, is an aggressive disease with an increased worldwide incidence. Pancreatic ductal adenocarcinoma (PDAC), ~90% of pancreatic malignancies, arises from pancreatic ducts. PC has a unique microenvironment hosting a heterogenous combination of cell populations, including immune cells and microbes. Microorganisms appear involved in every step of PC’s natural history, from creating a predisposing environment for *in situ* carcinogenesis to cell migration and metastasis. Biliary stent placement through endoscopic retrograde cholangiopancreatography (ERCP) can mitigate jaundice in PC patients but may alter the intestinal microbiota and contribute to tumor initiation and progression. Disruption of the antimicrobial barrier of the sphincter of Oddi, due to endoscopic sphincterotomy and stent insertion, promotes duodenal reflux, permitting bacterial colonization and biofilm formation. Although ERCP is the preferred drainage route, studies reported lower complication rate and reduced dysbiosis with percutaneous transhepatic biliary drainage (PTBD). The biliary microbiome in stented patients undergoing pancreaticoduodenectomy is altered, exhibiting higher levels of *Enterococci*, *Klebsiella*, and *Candida* species. The decision to place a biliary stent in PC patients should be carefully considered, given the potential for dysbiosis and its impact on therapeutic resistance. This underscores the need for further research into interventions that could modulate the microbiota, such as PTBD, probiotics or targeted microbial therapies.

## 1 Introduction

Pancreatic cancer is a highly lethal malignancy, with a poor prognosis that reflects late-stage diagnosis and the limited effectiveness of available treatments. Tobacco use, obesity, and a family history of pancreatic cancer are well-established risk factors, along with genetic mutations such as breast cancer gene 1 and 2 (BRCA1/2) and Kirsten rat sarcoma viral oncogene homolog (KRAS). Chronic inflammation, especially in conditions like pancreatitis, has also been implicated in increasing the risk of pancreatic cancer. However, despite these known risk factors, early detection remains elusive, and most patients are diagnosed at advanced stages when curative treatment is no longer an option.

Pancreatic ductal adenocarcinoma (PDAC), the most common form of pancreatic cancer, is resistant to chemotherapy and radiation, contributing to its high mortality rate. In the quest for novel treatment strategies and biomarkers for early detection, research into the role of the microbiome in cancer development has emerged as a promising field of investigation. The human microbiome, particularly the gut microbiome, consists of a complex community of bacteria, archaea, fungi, and viruses that co-evolve with the host.

The gut microbioma plays a crucial role in liver and pancreas health, influencing diseases such as non-alcoholic fatty liver disease (NAFLD), liver cirrhosis, porto-sinusoidal vascular disease (PSVD), pancreatitis, and pancreatic cancer (1, 2).

The microbial community has been implicated in various cancers, including colorectal, gastric, and hepatic cancers. Emerging evidence suggests that dysbiosis - an imbalance in microbial composition - may also contribute to pancreatic cancer. Microbial signatures in both gut and pancreas itself have been found to differ between healthy individuals and pancreatic cancer patients. Furthermore, the microbiome is thought to influence pancreatic cancer through various mechanisms, including immune modulation, chronic inflammation, and the production of microbial metabolites that impact tumor growth.

This review aims to provide a comprehensive overview of the current understanding of the microbiome’s involvement in pancreatic cancer development and progression. We will explore the links between specific microbial taxa and pancreatic cancer, outline potential mechanisms of action, and discuss how microbiome-based therapies could offer new avenues for treatment and early detection.

## 2 Review strategies and literature included

The literature search for this narrative review was conducted using the PubMed and Scopus databases. The search included articles published from January 2018 to January 2025 and was performed using combinations of the following keywords: “pancreatic cancer”, “biliary stent”, “dysbiosis”, “gut microbiota”, and “chemotherapy resistance”.

Articles were initially screened based on their relevance to the relationship between biliary drainage procedures, microbiota alterations, and pancreatic cancer development, progression, or treatment response. Both preclinical and clinical studies were considered, including observational studies, experimental studies, and relevant review articles.

Inclusion criteria were: (i) studies investigating the microbiome of the gut, bile, or pancreatic tissue in patients with pancreatic cancer; (ii) studies evaluating the impact of biliary stent placement or biliary drainage procedures on microbial composition; and (iii) studies addressing the role of microbiota in pancreatic cancer progression, immune modulation, inflammation, or chemotherapy resistance. Exclusion criteria included: (i) articles not written in English; (ii) studies unrelated to pancreatic cancer or biliary interventions; (iii) case reports with insufficient microbiological or mechanistic data; and (iv) abstracts without full-text availability.

After title and abstract screening, full texts of potentially eligible articles were assessed, and 66 studies published within the predefined time frame were selected for detailed analysis. Titles and abstracts were screened independently by the authors, and discrepancies were resolved through discussion.

In addition, seminal studies, landmark papers, published outside the selected time period were included to provide historical background and to support the biological and conceptual framework of the review. These additional references were not part of the structured selection process but were cited to ensure a comprehensive and contextual discussion of the topic.

**Figure 1:**
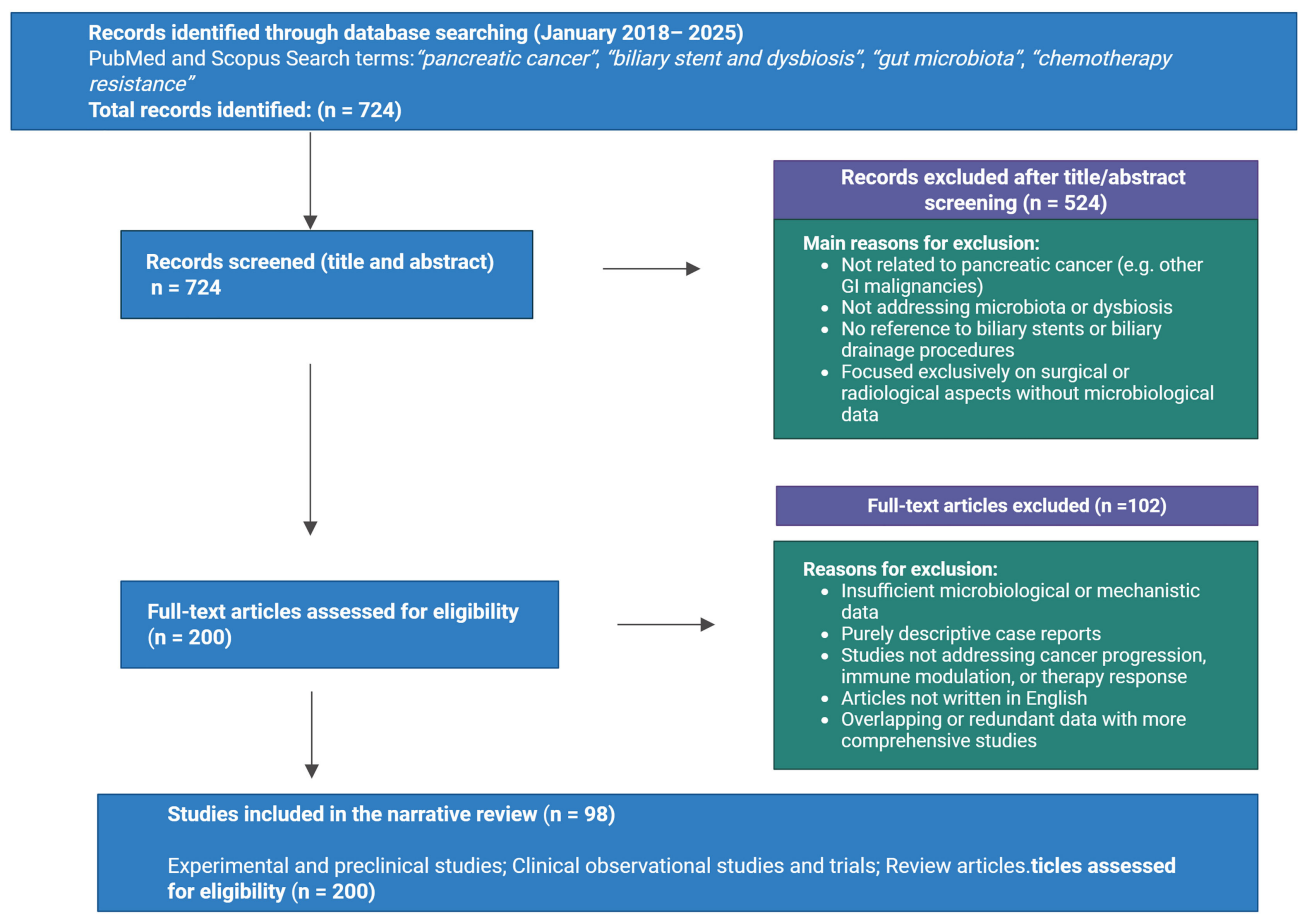
Literature search and article selection flow diagram. The diagram summarizes the main steps of database searching, screening, and selection of articles included in this narrative review. Both original studies and review articles were considered. The flow chart is provided for descriptive purposes and does not follow PRISMA guidelines, which are specific to systematic reviews.

Given the narrative nature of this review, no formal quality assessment or scoring system was applied. The literature search and article selection process is summarized in **Figure 1**. The flow diagram is intended to provide a descriptive overview of the study selection process and does not follow PRISMA guidelines, which are specific to systematic reviews.

## 3 The microbiome in a healthy pancreas

Historically, the pancreas was considered a sterile organ due to its position in the retroperitoneum and the production of digestive enzymes capable of destroying bacteria, whose presence was regarded as a sign of infection or disease. This belief began to change with advancements in DNA sequencing and microbial detection techniques (such as 16S rRNA sequencing), which have allowed researchers to identify microbial communities in areas previously thought to be sterile, such as the pancreas. Within the pancreas, there exists a small but distinct microbiome, primarily composed of bacteria commonly found in other parts of the gastrointestinal tract. These bacteria, although present in lower numbers compared to the gut, appear to play a role in maintaining the health and normal function of the organ. Certain bacterial phyla, particularly Proteobacteria, Firmicutes, and Bacteroidetes, have been consistently identified in healthy pancreatic tissue. The phylum Proteobacteria includes a variety of Gram-negative bacteria, some of which are known for their role in inflammation and metabolic processes. Firmicutes, on the other hand, include beneficial gut bacteria such as *Lactobacillus* and *Clostridium*, which may help balance immune responses within the pancreas. Bacteroidetes are abundant Gram-negative bacteria in the gut and may influence the local immune environment of the pancreas. In addition to bacteria, some evidence suggests the presence of fungal and viral components within the pancreatic microbiome, although these microorganisms have been less extensively studied (3).

The origin of the pancreatic microbiome is still the subject of ongoing research. One hypothesis is that bacteria reach the pancreas from the intestine, traveling through the biliary and pancreatic ducts, while many others believe it is possible through systemic circulation or as a result of endoscopic procedures involving stent placement. The presence of bacteria in the pancreas associated with the intestine supports the concept of microbial translocation. This theory aligns with the gut-pancreas axis, a concept suggesting a bidirectional relationship between the microbiomes of the gut and the pancreas (4).

In recent years, research has shown that the microbiome is not only involved in maintaining homeostasis but under conditions of dysbiosis it can also contribute to the development of diseases, such as pancreatitis, diabetes and cancer (5).

Microbiome dysbiosis has been implicated in carcinogenesis through several mechanisms, such as inflammation, genotoxicity, and modulation of the immune system.

Emerging evidence suggests that the normal microbiome can play a protective role in the pancreas, potentially influencing the development of pancreatic cancer. The human microbiome, particularly the gut microbiome, interacts with the immune system and modulates inflammation, which is critical in preventing or promoting cancer development (6).

In the following paragraphs the interplay between the commensal microbiota and the immune system function in homeostasis and pancreatic cancer will be discussed.

### 3.1 Immune modulation

The microbiome helps regulate the immune responses, including the balance between pro-inflammatory and anti-inflammatory signals.

Natural killer (NK) cells, effector lymphocytes of the innate immune system, exert an anti-tumor function due to their ability to migrate and infiltrate the tumor microenvironment (7, 8). Once activated, NK cells induce apoptosis of target cells through granule-mediated pathways. Additionally, NK cells secrete interferon-gamma (IFN-γ) and tumor necrosis factor-alpha (TNF-α), which trigger the activation and recruitment of other innate and adaptive immune cells. Previous studies have shown that tumor infiltration by NK cells and their subsequent activation are positively correlated with the survival of PDAC patients (9, 10). In advanced stages of PDAC, however, the antitumor effect of NK cells appears to be lost, as evidenced by the decrease in IFN-γ levels.

Yu et al., in their study conducted on murine models, demonstrated that the dysbiosis inhibits the infiltration and activation of intratumoral NK cells, resulting in increased progression of PDAC. NK cells are crucial for the progression of microbiota-mediated PDAC in immunodeficient and immunocompetent murine models, as antibody-mediated NK cell depletion in both models resulted in advanced tumors despite the absence of microbiota in mice treated with antibiotics. A healthy microbiome can support anti-tumor immunity by activating immune cells like T cells and NK cells, which may suppress pancreatic cancer growth (11).

The immune system plays a dual role in cancer, acting both as a barrier to tumorigenesis and, paradoxically, as a facilitator of tumor growth when dysregulated. The microbiome can influence this balance through its interactions with the immune system. In pancreatic cancer, dysbiosis can lead to immune suppression, promoting an immunosuppressive tumor microenvironment (TME). Specifically, bacterial metabolites and microbially derived molecules can interact with toll-like receptors (TLRs) on immune cells, promoting the recruitment of immunosuppressive cells such as regulatory T cells (Tregs) and myeloid-derived suppressor cells (MDSCs). MDSCs are immature myeloid cells able to suppress both innate and adaptive immunity with different mechanisms, promoting regulatory T cell development and simultaneously inhibiting the effector T cells and NK cells (12).

### 3.2 Inflammation control

Chronic inflammation is a known risk factor for pancreatic cancer. Oncogenic KRAS is the most common mutation driver in human PDAC, being present in over 90% of cases. There is a synergistic relationship between KRAS activation and inflammation, as KRAS expression is inherently highly pro-inflammatory (13, 14).

The gut microbiome produces metabolites, such as short-chain fatty acids (SCFAs), that have anti-inflammatory properties and may reduce chronic inflammation in the pancreas, lowering cancer risk. Indigestible carbohydrates (e.g., dietary fibers) are fermented by the gut microbiota, producing SCFAs such as acetate, propionate, and butyrate (15). Propionate and acetate are primarily produced by Bacteroidetes, while butyrate is produced by Firmicutes (16–18). Colonocytes use most of the butyrate as an energy source (19). SCFAs enter the liver through the portal vein (20), where propionate and acetate are metabolized to generate glucose and used as substrates in lipogenesis (19, 21). Due to their ability to reach various systemic tissues, SCFAs also affect immune system regulation, anti-inflammatory responses, blood pressure, and energy supply (22). Acetate can mitigate pancreatitis by offering protection against PDAC. Butyric acid can reduce the growth of PDAC cells in culture and promote differentiation. It has been demonstrated that hyaluronic acid conjugated with butyrate is cytostatic in cultured PDAC cells (23).

Butyrate has been described as possessing anti-inflammatory and antineoplastic properties: specifically, in the context of pancreatic cancer, butyrate has been found to exhibit “pro-differentiating, anti-proliferative, anti-invasive, pro-apoptotic” and chemo sensitizing effects in PDAC cell lines *in vitro* (24). Sodium butyrate also prevents the activation of pancreatic stellate cells, reducing the desmoplastic reaction in PDAC (25).

Farrow et al. reported that β4 expression is higher in more aggressive form of pancreatic cancer and found that sodium butyrate reduces integrin β4 expression in pancreatic tumor cells. Thus, by inhibiting β4 expression and tumor invasion, sodium butyrate may represent a promising strategy to counteract cancer progression (26).

### 3.3 Barrier function

A healthy microbiome helps maintain the integrity of the gut lining. If the gut barrier is weakened, harmful bacteria or endotoxins could translocate into the bloodstream, promoting systemic inflammation, which could affect pancreatic tissues and increase cancer risk. For example, *Porphyromonas gingivalis* and *Aggregatibacter actinomycetemcomitans*, which are linked to periodontal disease, have been associated with an elevated risk of PDAC. These pathogens can induce local inflammation, DNA damage, and immune suppression, facilitating tumor initiation and progression (27).

## 4 Microbiome and carcinogenesis

The role of the microbiome in cancer has been well-established in gastrointestinal cancers, particularly colorectal cancer. Bacteria such as *Fusobacterium nucleatum* and *Helicobacter pylori* have been directly linked to tumorigenesis through mechanisms involving chronic inflammation, immune evasion, and the production of carcinogenic metabolites (28, 29).

Given the close anatomical and functional relationship between the pancreas and the gut, it is plausible that the microbiome could also influence the development of pancreatic cancer.

The normal microbiome varies between individuals and body sites, influenced by local conditions, host genetics, diet, antibiotic consumption, and lifestyle. In healthy conditions, commensal microorganisms interact symbiotically with the host. Dysbiosis, a disruption of this existing balance, can contribute to the pathogenesis of many diseases, including cancer (30). Nejman et al. demonstrated that each tumor type has a distinct microbial composition (31). Geller et al. compared pancreatic tumor tissues and normal pancreatic tissues, identifying bacterial DNA in 76% of tumors and in 15% of normal tissues (32). They identified Gammaproteobacteria as the most common taxa, including members of the Enterobacteriaceae and Pseudomonadaceae families, with *Pseudomonas*, *Citrobacter*, *Klebsiella*, *Streptococcus*, and *Acinetobacter* as the genera with the highest relative abundances. These findings are consistent with those reported by Nejman et al., demonstrating that over 60% of pancreatic tumors were positive for bacterial DNA. Consequently, it was also observed that bacterial DNA profiles were similar between the pancreas and duodenum of the same subjects, further supporting the migration of bacteria from the gut to the pancreas (33). These results suggest bacterial reflux from the duodenum to the pancreas through the major/minor papilla and the pancreatic duct, as Proteobacteria dominate the normal duodenal microbiome (31, 32). Nejman and colleagues also demonstrated that microbial populations present in tumor tissues of various cancer types are similar to those in adjacent normal tissues (31), leading to the hypothesis that the tumor microbiome evolves from microbes existing in normal tissues.

Pushalkar et al. demonstrated that the gut microbiome in patients with PDAC showed reduced bacterial diversity and an overrepresentation of certain bacterial genera, such as Proteobacteria and Firmicutes (34). The study also highlighted that these microbial changes led to alterations in the immune microenvironment, including the suppression of anti-tumor immune responses. For instance, *Fusobacterium nucleatum*, implicated in colorectal cancer, has also been found in pancreatic tumors. Furthermore, the presence of certain bacteria in pancreatic tissue has been correlated with patient prognosis and a reduced response to immunotherapy. A study by Riquelme et al. found that the dysbiosis within pancreatic tumors was associated with worse survival outcomes, and tumor cells harboring specific bacterial taxa were less responsive to immune checkpoint inhibitors, suggesting that the microbiome can influence the effectiveness of cancer treatments (35).

Finally, bile contamination, mainly related to biliary stenting and endoscopic procedures, was significantly found in patients affected by PDAC. The more represented species in bile samples (collected during endoscopic procedures or surgery) were *Escherichia coli* and different species of *Pseudomonas*, *Enterobacter*, and *Enterococcus* (36–39).

Furthermore, the presence of these microorganisms can interfere with normal cellular functions through the production of microbial metabolites and toxins. Bacterial metabolites like LPS can activate TLRs on pancreatic cells, leading to downstream signaling that promotes oncogenic pathways. Similarly, fungal components such as β-glucans, which are polysaccharides found in the cell walls of fungi, can activate the complement pathways. This activation promotes enhanced inflammatory responses, which, in turn, may create a microenvironment favorable to tumor progression (40).

## 5 STENT and microbiome

Biliary stents, commonly used to manage obstructive jaundice caused by biliary strictures or tumors, represent another critical factor in the pancreatic ecosystem.

Physiologically, bile flows from the common bile duct (CBD) into the duodenum in only one direction. This process is controlled by the sphincter of Oddi, which prevents duodenal contents from refluxing into the biliary tree. When a biliary stent is placed endoscopically through the papilla of Vater, it bypasses the sphincter of Oddi. This eliminates its barrier function and creates a continuous connection between the duodenum and the biliary system. Loss of the sphincter’s ability to function properly disrupts the normal pressure gradient that pushes bile into the system. Under these circumstances, temporary increases in pressure in the duodenum can exceed the pressure in the bile ducts, causing duodenal fluid to reflux into the CBD. The stent also facilitates reflux by acting as a low-resistance channel and providing a surface that aids reverse transport.

**Figure 2:**
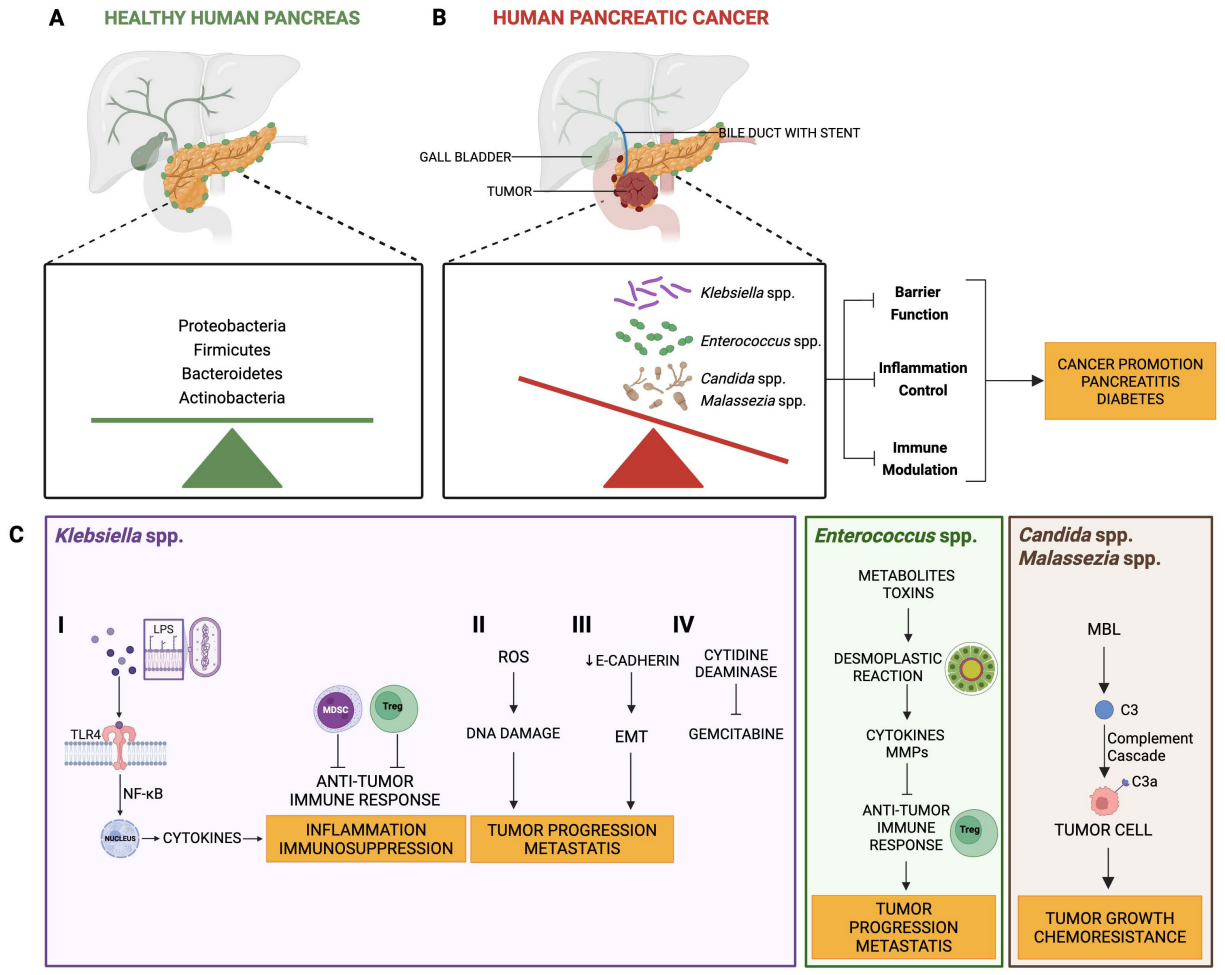
Modification of the pancreatic microbiome in patients with biliary stent. **(A)** The pancreas of healthy individuals is colonized by a wide range of bacteria in a homeostatic balance. **(B)** The presence of the stent would favor the reflux of bacteria from the intestine to the pancreatic tissue through the Vater papilla. This migration could promote a dysbiosis in favor of bacteria such as *Klebsiella*, *Enterococci*, *Candida*, *Malassezia*, etc. These are responsible for an increase in inflammation at the pancreatic level with possible development or progression of a tumor. **(C)** The main mechanisms of action of the predominant bacteria in patients with pancreatic cancer undergoing stent placement are illustrated. Different pathways ultimately converge at a common point: tumor growth, metastasis and resistance to chemotherapy. LPS, bacteria lipopolysaccharide; TLRs, toll-like receptors; NF-κB, nuclear factor kappa B; Tregs, regulatory T cells, MDSCs, myeloid-derived suppressor cells, MMPs, matrix metalloproteases; MBL, mannan-binding lectin.

While stents are effective at alleviating symptoms of bile duct obstruction, they also create a path for bacteria to migrate from the duodenum into the biliary system and, potentially, the pancreas and canthus altering the microbial environment. Bacteria can adhere to the stent surface, forming biofilms that are difficult to eradicate, even with antibiotic treatment. Stent-related infections often result from biofilm-forming bacteria like *Escherichia coli*, *Klebsiella pneumoniae*, and *Enterococcus faecalis* (**Figure 2** and **Table 1**). These bacteria may also enter the pancreatic ductal system, exacerbating pancreatitis or contributing to secondary infections.

**Table 1:** Bacterial species and pathogenic mechanisms implicated in biliary stent–related infections in pancreatic cancer and supporting references.

Pathogen	Main mechanisms involved in pathogenesis	References
*Klebsiella* spp	- Induction of chronic inflammation (↑ IL-6, TNF-α) leading to pro-tumorigenic microenvironment. - Activation of TLR4/NF-κB signaling via LPS → immune suppression and recruitment of Tregs and MDSCs. - Production of ROS and induction of DNA damage, facilitating KRAS and TP53 mutations. - Promotion of epithelial–mesenchymal transition and metabolic reprogramming (Warburg effect). - Expression of cytidine deaminase → inactivation of gemcitabine.	(49– 61)
*Enterococcus* spp.	- Production of lipoteichoic acid → activation of NLRP3 inflammasome → ↑ IL-1β/IL-18. - Promotion of desmoplastic reaction via cytokines, chemokines and MMPs → stromal fibrosis and drug resistance. - Upregulation of PD-L1, promotion of immune system evasion. - Association with increased Enterobacteriaceae after chemotherapy and with chemoresistance.	(62–71)
*Candida* spp.	- Trigger of inflammatory responses and Th17 activation. - Production of metabolites promoting tumor proliferation. - Contribution to dysbiosis and reduction in chemotherapy efficacy.	(45, 46, 71, 75)
*Malassezia* spp.	- Activation of complement cascade via mannose-binding lectin. - Increase in C3a/C5a → promotion of proliferation, EMT, metastasis. - Recruitment of MDSCs and Tregs → immunosuppressive tumor microenvironment.	(73, 74)

DNA, deoxyribonucleic acid; EMT, epithelial-mesenchymal transition; IL-1β, interleukin1β; IL-6, Interleukin-6; IL-18, interleukin-18; KRAS, Kirsten rat sarcoma viral oncogene homolog; MDSCs, myeloid-derived suppressor cells; MMPs, metalloproteinases; NLRP3, NLR family pyrin domain containing 3; NF-κB, nuclear factor kappa B; PDL-1, Programmed Death-Ligand 1; ROS, reactive oxygen species; TLR-4, toll-like receptor-4; TNF-α, tumor necrosis factor-alpha TP53, tumor protein 53; Tregs, regulatory T cells.

In patients with pancreatic cancer, biliary stents can further complicate the microbiome landscape. Given the immunosuppressive nature of pancreatic tumors, the altered microbial environment resulting from stent placement may contribute to tumor progression by promoting chronic inflammation or producing microbial metabolites that support cancer cell survival.

Blanco-Míguez et al. analyzed the microbial species present on biliary stents from 56 patients collected either during endoscopic procedures or during pancreaticoduodenectomy surgeries (41). Microbial DNA was extracted from cultivated biofilms. Indeed, in more than 80% of the samples, only three species were detected: *Streptococcus anginosus*, *Escherichia coli*, and *Enterococcus faecalis*. They then sought to investigate the potential bodily site of origin of the bacteria colonizing the stents. *Escherichia coli*, *Klebsiella pneumoniae*, and various lactic acid bacteria, were commonly the dominant species on the stents. In the evaluation of bile samples as a potential site of origin, only the first two bacteria were found with similar prevalence (42, 43). Instead, almost half of the prevalent species in the stents are common members of the human oral microbiome and, when present in the intestine, are predominantly associated with various diseases such as colorectal cancer, inflammatory bowel disease, atherosclerotic cardiovascular disease, and cirrhosis. No difference was detected in the type of stent, whether metallic or plastic.

Stents can also frequently become obstructed, preventing the flow of bile into the intestine, further altering the bacterial population. Cacaci et al., analyzed bile and the corresponding stent samples to determine the microbial population primarily involved in the stent occlusion process (44). The most common aerobic bacteria isolated from bile and stent samples were Gram-positive *Enterococcus* species, particularly *Enterococcus faecalis* and *Enterococcus faecium*, and Gram-negative bacteria *Escherichia coli* and *Klebsiella pneumoniae* (45, 46). Among yeasts, *Candida albicans* was found to be the predominant species (45, 46). The anaerobic bacteria identified varied among different studies. A study by Leung et al., reported the early attachment of *Clostridium perfringens*, *Clostridium bifermentas*, and *Bacteroides fragilis* to stents as a critical step toward the stent occlusion process (47). Another study reported the presence of *Bacteroides* spp, *Prevotella* spp., and *Veillonella* spp. as predominant anaerobic species (46), while another research identified *Fusobacterium* spp. and *Veillonella* spp. as the most observed anaerobic species (41).

Moreover, the role of pancreatic microbiota in tumor progression is evident even in cystic precursors of pancreatic cancer, such as intraductal papillary mucinous neoplasms (IPMNs). Halimi et al., cultured bacteria from these cystic lesions, identifying species such as *Klebsiella pneumoniae*, *Enterococcus faecalis*, and *Granulicatella adiacens*, which can survive inside pancreatic cells and cause DNA damage, suggesting a role in tumor progression (48).

### 5.1 The role and pathogenic mechanism of *Klebsiella* in pancreatic cancer

*Klebsiella* spp. are facultative anaerobic Gram-negative rods that are part of the normal flora of the gastrointestinal tract but can become pathogenic in immunocompromised individuals. Studies have shown that patients with pancreatic cancer have an overrepresentation of *Klebsiella* in the pancreatic and gut microbiome. The migration of *Klebsiella* from the gastrointestinal tract into the pancreatic duct may be facilitated by biliary stents, leaky gut syndrome or altered mucosal barriers, a common phenomenon in chronic pancreatitis, a well-established risk factor for pancreatic cancer. Once *Klebsiella* reaches the pancreatic tissue, it may contribute to tumorigenesis and tumor progression (49).

Chronic pancreatitis and inflammation are well-known established risk factors for PDAC. *Klebsiella* can induce an inflammatory response in pancreatic tissue, leading to the activation of pro-inflammatory cytokines such as Interleukin-6 (IL-6) and TNF-α, which are involved in promoting tumor cell proliferation and survival. This inflammatory microenvironment can facilitate mutations in oncogenes such as KRAS and tumor suppressor genes like TP53, thereby promoting the onset of pancreatic cancer (50). Moreover, *Klebsiella* can evade immune detection through various mechanisms, including the production of a polysaccharide capsule and lipopolysaccharides (LPS), which interact with TLRs, primarily TLR4. Activation of TLR4 by LPS can trigger signaling pathways such as Nuclear Factor kappa B (NF-κB), leading to the production of cytokines that drive immune suppression and promote a pro-tumorigenic microenvironment (**Figure 2A**). These cytokines storm not only results in chronic inflammation but also promote the recruitment of cells such as Tregs and MDSCs that further suppress the anti-tumor immune response. In pancreatic cancer, an immunosuppressive microenvironment enables tumor cells to evade immune surveillance, thereby fostering uncontrolled proliferation (51).

The tumor microenvironment is characterized by metabolic alterations that support tumor cell proliferation. *Klebsiella*, through the production of bacterial metabolites such as SCFAs and toxins, can reprogram the metabolism of pancreatic epithelial cells. This reprogramming shift cellular metabolism toward glycolysis (the Warburg effect), a hallmark of tumor cells, providing them with the energy and biosynthetic precursors necessary for rapid growth. Moreover, the metabolic changes induced by *Klebsiella* can promote epithelial-mesenchymal transition (EMT) (**Figure 2C**), a critical step in cancer invasion and metastasis (52). EMT is a physiological process occurring in multicellular organisms, characterized by changes in epithelial cells that lose their differentiated phenotype to acquire *de novo* mesenchymal traits (53).

The induction of the EMT process can be mediated and sustained by interactions between pathogenic bacteria and the epithelium. In this sense, the ability of certain entero-adherent bacteria to trigger and maintain a chronic inflammatory environment is noteworthy, as it is a prerequisite for the subsequent potential development of EMT-like phenotypes (54). Besides *Klebsiella*, other bacteria such as *Helicobacter pylori* and *Enterococcus faecalis* can also cause intracellular stress with tissue/organ damage (55, 56) and may promote the acquisition of malignant phenotypes (57). *Klebsiella* also produces a series of virulence factors, including siderophores (iron-scavenging molecules), which are essential for bacterial survival in nutrient-poor environments such as tumors. These factors can alter cellular metabolism, increase oxidative stress, and promote DNA damage, contributing to the mutational burden in pancreatic cells. Reactive oxygen species (ROS) produced as a result of bacterial infection further exacerbate cellular damage, increasing the likelihood of tumorigenic mutations (**Figure 2B**) (58, 59). Oxidative stress promotes genomic instability, which can accelerate the accumulation of mutations in key regulatory genes such as KRAS, TP53, and mothers against decapentaplegic homolog 4 (SMAD4), commonly mutated in PDAC. This is a frequent event in the microenvironment of infected tissues and triggers pathways through the induction of hypoxia-inducible factor 1-alpha (HIF-1α), leading to the activation of histone deacetylase 3 (HDAC3), which is essential for EMT-like processes and metastasis (60). Inflammation induced by *Klebsiella* can also exacerbate these mutational events by impairing DNA repair mechanisms.

Finally, the presence of *Klebsiella pneumoniae* is associated with reduced progression-free survival and larger tumor sizes, indicating their role in exacerbating the disease state (61).

### 5.2 The role and pathogenic mechanism of Enterococcacae and Enterobacteriaceae in pancreatic cancer

*Enterococcus* species, predominantly *Enterococcus faecalis* and *Enterococcus faecium*, are Gram-positive bacteria that are part of the normal human gut flora but can also act as opportunistic pathogens. Disruption of the intestinal barrier or the presence of biliary stents potentially allows *Enterococcus* to colonize the pancreas. Once in the pancreatic microenvironment, *Enterococcus* can secrete metabolites and toxins that alter the local immune response and extracellular matrix, facilitating tumor growth and metastasis. The presence of bacteria in the pancreas has been shown to induce fibrosis and desmoplastic reaction, which is a hallmark of pancreatic cancer. The desmoplastic reaction is a fibrotic response characterized by a large amount of dense extracellular matrix (ECM) and a dense fibrous stroma (**Figure 2**). The fibrotic stroma surrounding the tumor comprises various components, including myofibroblasts, collagen, and other extracellular matrix molecules. Multiple processes are involved in fibrosis, largely associated with the upregulation of various cytokines, chemokines, matrix metalloproteinases, and other growth factors that promote tumor growth and metastasis. Fibrosis is also associated with the recruitment of immunosuppressive cells, such as Tregs, which function to suppress anti-tumor immunity. Additionally, dense fibrosis limits blood flow to tumor cells, which may contribute to drug resistance (62).

In addition to inducing immune tolerance, *Enterococcus* can promote the expression of immune checkpoint molecules such as programmed death-ligand 1 (PD-L1) in pancreatic cancer cells, further inhibiting anti-tumor immunity.

Maekawa et al., recently reported that most PDAC patients have subclinical chronic pancreatitis (CP) in the pancreatic tissue surrounding tumors (63).

*Enterococcus* species have been shown to induce inflammatory responses through several mechanisms, one of which is guided by lipoteichoic acid (LTA), an important etiological agent of Gram-positive bacteria, regarded as the counterpart of LPS in Gram-negative bacteria (64). LTA can induce inflammatory responses by triggering pro-inflammatory cytokines and chemokines in the host (65, 66), involving the activation of the inflammasome 3 (NLRP3). Inflammasomes are cytosolic protein complexes that mediate innate immunity by releasing IL-1β and IL-18 (67). Inflammasome activation is induced by two pathways: the canonical caspase-1-mediated pathway and the non-canonical caspase-4-mediated pathway, caspase-5 (68). Pathogen-derived ligands such as LTA can induce pro-IL-1β expression and NLRP3 activation. This complex reaction involves the cleavage of caspase-1 into the active form responsible for the mature secretion of IL-1β as the final step (69).

Chronic activation of the NLRP3 inflammasome has been implicated in pancreatic tumorigenesis by promoting cellular proliferation and inhibiting apoptosis (70). In a study by Pushalkar et al., antibiotic treatment that altered gut microbiota, including a reduction in *Enterococcus*, led to a decrease in pancreatic tumor burden and improved response to immune checkpoint inhibitors (34). These findings highlight the potential of targeting gut microbiota, including Enterococcus, to modulate tumor progression.

Patient receiving combined neoadjuvant chemotherapy with Gemcitabine and Paclitaxel had higher levels of Enterobacteriaceae, a family of bacteria that has been implicated in conferring resistance to chemotherapy agents like Gemcitabine, compared to those treated with Gemcitabine alone or no chemotherapy (40, 71). Biliary stent placement is correlated with increased relative abundances of Enterobacteriaceae, suggesting that biliary stents can alter the microbial environment in a way that may support tumor progression and impact the effectiveness of chemotherapy (71).

Specific bacteria, such as *Enterotoxigenic Bacteroides fragilis* (ETBF) and *Fusobacterium nucleatum*, enhance tumor growth by activating pathways like Wnt/ß-catenin through interactions with E-cadherin. These bacteria promote epithelial cell proliferation and tumorigenesis by driving the transcription of oncogenes such as c-Myc. Moreover, microbial metabolites, such as butyrate, exhibit a dual effect in cancer progression. On one hand, they can suppress cancer cell proliferation by influencing epigenetic mechanisms, on the other hand, disruptions in their metabolic processes may trigger apoptosis in healthy cells, thereby indirectly facilitating tumor development (72).

### 5.3 Fungal dysbiosis contributes to the progression of pancreatic cancer

Fungal dysbiosis contributes to the progression of pancreatic cancer, particularly in patients with biliary stents. The study found a substantial increase in fungal presence within pancreatic tumors compared to normal tissue, with *Malassezia* being notably prevalent (73).

*Malassezia* spp. are linked to worse outcomes and facilitate tumor growth by activating the complement system through mannose-binding lectin (MBL), leading to inflammation and immunosuppression. This process involves components like C3a and C5a, which promote cell proliferation and EMT, and recruit MDSCs and Tregs, creating an immunosuppressive environment (**Figure 2**) (73, 74).

Fungal infections, specifically those caused by *Candida* spp., are able to promote cancer development. *Candida* spp., as opportunistic pathogens, inhabit the mucosal epithelium and cause oral lesions. *Candida albicans*, the dominant species, contribute to tumor progression in pancreatic cancer patients with biliary stents through the production of byproducts that trigger inflammation, induce the Th17 response, and engage in molecular mimicry, promoting cancer development and proliferation (**Figure 2**) (75).

## 6 Chemotherapy-induced dysbiosis and resistance in pancreatic cancer patients

Chemotherapy drugs can alter the gut microbiota composition, reducing beneficial bacteria like *Lactobacillus* and *Bifidobacterium* and increasing harmful bacteria such as *Escherichia coli* and *Staphylococcus*. This shift in microbiota composition is associated with an activated inflammatory pathway and impaired barrier function, making the host more vulnerable to pathogens (76). Chemotherapy can alter the gut and biliary microbiome, reducing microbial diversity and promoting the growth of resistant strains. This dysbiosis not only leads to increased susceptibility to infections but also has been shown to contribute to chemotherapy resistance (61).

Dysbiosis can affect the absorption and metabolism of chemotherapeutic agents, potentially leading to decreased efficacy and increased resistance (77). For instance, it was noted that the gut microbiota might contribute to resistance against chemotherapeutic drugs like gemcitabine by modulating the immune environment.

Interestingly, *Klebsiella pneumoniae* has been found to interfere with the efficacy of gemcitabine by expressing enzymes such as cytidine deaminase, which inactivates the drug (**Figure 2C**), highlighting the critical role of microbiota in influencing treatment outcomes (61, 78). Reducing such bacterial populations can enhance survival and response to treatment, indicating that dysbiosis can contribute to chemotherapy resistance (74). Furthermore, bacteria such as Gammaproteobacteria, which can metabolize chemotherapeutic agents like Gemcitabine, may contribute to chemoresistance, thus complicating treatment outcomes (**Figure 2**) (71, 72). Additionally, the authors investigate the interplay between chemotherapy and fungal dysbiosis, revealing that antifungal treatment can enhance the efficacy of gemcitabine chemotherapy (71, 72). *Candida albicans* infection might lead to dysbiosis, affecting the gut microbiota and potentially diminishing the efficacy of chemotherapy (75).

Dysbiosis can lead to an increase in Tregs and MDSCs, both of which suppress the immune system’s ability to attack tumor cells. This immune modulation can create a protective niche for cancer cells, enabling them to survive and proliferate despite chemotherapy (71).

Biliary stent placement is correlated with increased relative abundances of Enterobacteriaceae, a family of bacteria that has been implicated in conferring resistance to chemotherapy agents like gemcitabine, suggesting that biliary stents can alter the microbial environment in a way that may support tumor progression and impact the effectiveness of chemotherapy (40). The prolonged use of stents and exposure to antibiotics for managing stent-related infections can also select for resistant microbial populations, contributing to a cycle of dysbiosis and therapeutic resistance (71). The resistant bacterial strains identified (e.g., resistant to ampicillin-sulbactam, piperacillin-tazobactam, ciprofloxacin, and imipenem) due to preoperative biliary drainage (PBD) suggest that patients undergoing chemotherapy might experience reduced efficacy and increased resistance to these treatments due to the pre-existing resistant bacterial environment (79). Indeed, in patients undergoing chemotherapy, there is an increased risk of biliary sepsis, and the microbial organisms isolated from these stents showed high resistance rates to certain antibiotics, indicating that dysbiosis can contribute to chemotherapy resistance​​ (80).

## 7 Use of probiotics, fecal microbiota transplantation, immunotherapy and transhepatic stents to prevent or reduce dysbiosis

### 7.1 Probiotics and prebiotics

**Figure 3:**
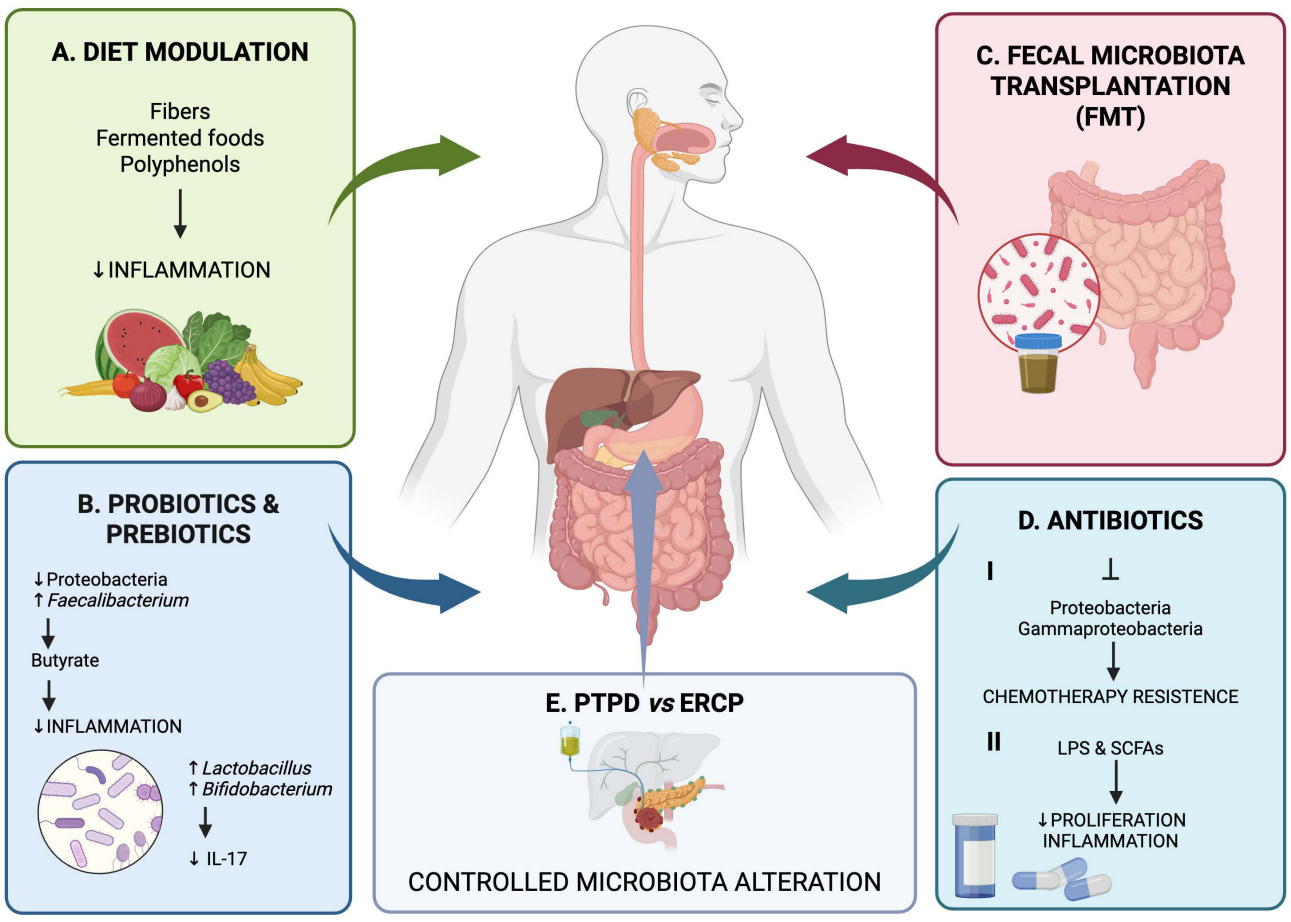
Understanding the interaction between the pancreas microbiome and biliary stents opens the door to microbiome-targeted therapies. **(A)** Consuming fibers, fermented foods, and polyphenols can help regulate gut microbiota and reduces inflammatory responses. **(B, C)** Probiotics, prebiotics, or microbiota transplants could potentially be used to restore a healthy microbial balance in patients with stents, especially in those suffering from recurrent infections or pancreatitis. Modulation of the intestinal microbiota through probiotics could positively influence tumor progression and improve response to oncological therapies. Probiotics promote the growth of beneficial bacteria, such as *Faecalibacterium prausnitzii*, which produce butyrate, a molecule with anti-inflammatory effects. Some probiotic strains, such as *Lactobacillus* and *Bifidobacterium*, can improve the anti-tumor immune response by increasing T cell activity and modulating the expression of pro-inflammatory cytokines (e.g. IL-17). Probiotic therapy or fecal microbiota transplantation (FMT) is being studied to restore a balanced microbial community, improving response to immune checkpoint inhibitors such as anti-PD-1 and anti-PD-L1. The use of antibiotics to modulate the microbiome in pancreatic cancer patients is an emerging strategy based on the ability to alter microbial communities that influence tumor progression. **(D)** Antibiotics can eliminate specific bacteria that promote tumor growth, such as certain species of Proteobacteria and Gammaproteobacteria. For example, these bacteria can metabolize drugs such as gemcitabine, rendering it ineffective. Reducing bacterial dysbiosis can improve the immune response. Preclinical studies have shown that abrogation of the gut microbiota with antibiotics can increase CD8^+^ T cell infiltration and reduce immunosuppressive cytokines such as IL-10 and IL-17. Antibiotics can indirectly influence the tumor microenvironment by modulating microbiota-derived metabolites, such as short-chain fatty acids and lipopolysaccharides (LPS), which influence proliferation and inflammation. **(E)** Furthermore, the choice of some biliary drainage procedures such as, PTBD vs ERCP could control the alteration of the microbiome.

Modulation of the intestinal microbiota through probiotics could positively influence tumor progression and improve response to oncological therapies. Probiotics can reduce the presence of pathogenic bacteria, such as Proteobacteria, which are often associated with dysbiosis in patients with pancreatic cancer. They promote the growth of beneficial bacteria, such as *Faecalibacterium prausnitzii*, which produce butyrate, a molecule with anti-inflammatory effects (**Figure 3**) (5, 81).

Studies showed that *Lactobacillus* can enhance the anti-tumor effects of gemcitabine in pancreatic cancer models (82). Another study further demonstrated that probiotics could mitigate the side effects of gemcitabine by restoring a favorable microbiota (83). Additionally, supercharged NK cells treated with probiotics were able to inhibit the growth of pancreatic tumors through direct lysis and differentiation in humanized-BLT mice (84). The anti-tumor effects of probiotics are thought to be mediated through the MAPK-p38 and TGF-β signaling pathways (85, 86).

Evidence suggests a strong gut-pancreas connection, leading to interest in using probiotics, prebiotics, symbiotics (a combination of probiotics and prebiotics), postbiotics and other microbiome-based therapies to improve outcomes.

A diet rich in fiber, polyphenols and fermented foods can support a more balanced microbiota and reduce systemic inflammation, indirectly improving oncological outcomes.

The preoperative administration of probiotics, prebiotics, and symbiotics effectively reduces postoperative infections, resulting in a decreased inflammatory response, morbidity, and hospital stay (87).

The administration of probiotics in patients with biliary stents can have a double function. Probiotics have both a direct interaction with the immune system and an indirect effect since they secrete a variety of probiotic-derived molecules (**Figure 3**). These latter are the competence and sporulation factor (CSF), the inorganic polyphosphates, the ferrochrome, and other peptides, including P75 and P40, which have been demonstrated to be associated with anticancer properties (88). Different mechanisms and pathways can be employed by these molecules. CSF is a quorum-sensing pentapeptide and it has the capacity to stimulate the upregulation of heat shock proteins. Moreover, it further activates the epithelial cell survival pathway of protein kinase B/Akt and p38 MAP kinase through the organic cation transporter 2, which has a cytoprotective effect (88).

### 7.2 Fecal microbiota transplantation

According to the idea that biliary stents modify the tumor microbiota, it may be considered the possibility of changing the gut microbiota through Fecal microbiota transplantation (FMT) (73). Initially, FMT was developed to fight against *Clostridium difficile* infection (CDI). Actually, the role of FMT to treat severe CDI was at first described by Eiseman and colleagues in 1958 in a case series of four patients with pseudomembranous enterocolitis cured with this new method (89). FMT involves transplanting fecal human microbiota from healthy individuals into the gastrointestinal tract of patients to reconstruct the normal and functioning intestinal microbiota (90). Anyway, Neemann et al. first established the link between FMT and cancer treatment in 2012 in a patient with acute lymphocytic leukemia (91). Subsequently, this therapy was implemented in the treatment of numerous other hematological malignancies. Although clinical trials concerning FMT to cure or prevent cancer patients are still in the early stages, at the present time, FMT has demonstrated its efficacy in various types of complications during anti-cancer treatment (92).

Indeed, it has been proved that microbiota from human FMT that is administered to murine PDAC models can be detected in PDAC microbiota post-transplantation, albeit in small percentage-less than 5% (35). *De facto*, Riquelme et al., in their innovative study, have shown that patients with long term survival - less aggressive - PDAC have higher tumor bacteria diversity, and the species found are *Pseudoxanthomonas, Streptomyces, Saccharopolyspora* and *Bacillus clausii.* Indeed, *in vitro*, through human-into-mice FMT experiments, the researchers can modulate the tumor microbiota and influence tumor growth and tumor immune infiltration, limiting the effects of *Enterococci, Klebsiella* and *Candida*, which are common in patients with biliary stents (35, 71). The latter may have a pivotal role since recent studies have suggested that a high level of microbial diversity in gastrointestinal microbiota relates to favorable treatment outcomes influencing chemotherapy and immunotherapy with anti-Cytotoxic T lymphocyte-associated antigen-4 monoclonal antibody and anti-programmed cell death-ligand 1 (PD-L1) (93).

However, although the increased microbial variety may have an immunoregulatory effect, its role in the antitumor response remains unclear. Probably, the presence of *Saccharopolyspora* spp., which is abundant in less severe PDAC, may promote the development of a pro-inflammatory microenvironment through the recruitment of inflammatory cells and the secretion of IFN-γ, which is mediated by cytokines and chemokines. Nevertheless, its function in PDAC has yet to be investigated more in detail.

Moreover, it is important to understand which are the temporal changes in the composition of the PDAC microbiota over time after FMT, as well as whether a single course of FMT has enduring effects on the PDAC (94). In conclusion, it is relevant to learn the -omics based field to rearrange the host microbiota since bacterial signatures can be useful to distinguish between cancer and non-cancer phenotypes, as well as between cancer types and stages because it would be unwise to blindly promote or eradicate a specific bacterial subspecies without knowing the reaction of the whole microbiota. The scientific community is awaiting the results of the PANDEMIC study (NCT04274972), a prospective, observational, cohort study that is currently in progress. It aims to evaluate the qualitative and quantitative analysis of the pancreatic microbiome in human patients with PDAC who are undergoing pancreaticoduodenectomy and intraoperative lesion sampling. In particular, the correlation between post-operative complications and bile, oral, and rectal microbiome samples will be investigated (PANDEMIC study).

### 7.3 Fecal microbiota transplantation and immunotherapy

FMT has recently garnered attention for its potential to enhance immune checkpoint blockade therapies and overcoming drug resistance (95). Preclinical models have demonstrated that FMT from cancer patients into germ-free (GF) mice can replicate the responder or non-responder phenotype to anti–PD-1 therapy. In these models, GF mice receiving fecal material from patients who responded to PD-1 therapy showed improved responses to therapy, while mice receiving non-responder fecal material did not. Remarkably, additional FMT from responder patients was able to reverse the non-responder phenotype in mice (93, 96). In this case the gut microbiome of long-term pancreatic cancer survivors showed higher diversity and the presence of beneficial bacterial species such as *Pseudoxanthomonas*, *Saccharopolyspora*, and *Streptomyces*. FMT from these patients into mice significantly reduced tumor growth, suggesting a potential protective effect of certain microbial communities against tumor progression (97). Moreover, clinical observations have shown that patients treated with broad-spectrum antibiotics exhibited reduced efficacy to anti–PD-1 therapy, highlighting the critical role of a healthy microbiome in supporting immunotherapy (96). Several clinical trials are currently underway to evaluate the potential of FMT in enhancing checkpoint blockade therapies in many tumors, such as metastatic melanoma (98). While the initial results are promising, caution is advised due to the risk of transferring pathogenic microorganisms through FMT, as highlighted by a safety bulletin from the US FDA (98). A recent study found that only 3% of potential FMT donors met the stringent criteria for clinical use, underlining the need for high standards in donor selection (99). Additionally, identifying patients who fail anti–PD-1 therapy due to microbiota-related defects remains a challenge. Resistance to therapy may not only stem from microbial composition but also from tumor cell-intrinsic factors and genetic variations in immune regulatory genes (100–103). Future protocols will need to rigorously screen FMT donors for pathogenic bacteria, parasites, and multidrug-resistant strains to minimize these risks.

### 7.4 Bacterial therapy

Another therapeutic avenue being explored is the administration of specific bacterial strains known to boost anti-tumor immunity. For example, Bifidobacteria species have demonstrated the ability to enhance T cell responses and improve the efficacy of anti–PD-1 therapies (104). A strain of *Bifidobacterium animalis lactis* (EDP1503) has been developed for clinical testing, where it acts at the level of the small intestine, triggering a broad immune response without needing to colonize the gut. This strain has shown promise in preclinical models, where it promotes inflammatory cytokines production, T cell activation, and NK cell responses (104).

## 8 Types of biliary drains and microbiome

Patients with pancreatic head cancer often suffer from obstructive jaundice, necessitating preoperative biliary stenting, especially in the context of neoadjuvant therapy.

The type of stent used for PBD also influences postoperative outcomes. Self-expandable metal stents have been shown to offer better patency and lower infection rates compared to plastic stents. A randomized trial by Soderlund et al. confirmed these findings, reporting a median better patency for metal stents (105). In contrast, plastic stents were associated with an increased incidence of cholangitis and greater bacterial resistance due to more frequent antibiotic use (106).

In an evidence based-review Dorcaratto et al., compared two methods of PBD in jaundiced patients undergoing pancreaticoduodenectomy (PD): PTBD and endoscopic biliary drainage (EBD). The analysis evaluates which method is superior in reducing complications, such as infection or liver function issues, before surgery (107). The review consolidates data from several studies to assess the safety, efficacy, and outcomes of each method in patients preparing for PD, with particular focus on the risks of cholangitis, stent dysfunction, and overall mortality rates. The review concludes that PTBD may offer certain advantages over EBD in the management of jaundiced patients awaiting PD. Specifically, PTBD is associated with a lower incidence of cholangitis and stent dysfunction, although the overall mortality rates between the two methods were similar. Moreover, in terms of bacterial load, the median was significantly higher in patients who underwent endoscopic biliary drainage, with a significantly higher Proteobacteria load. In addition, patients with biliary drainage showed a higher specific diversity.

PTBD is often associated with a reduced bacterial load due to continuous external drainage, potentially lessening microbiome alterations compared to stents. However, PTBD has its own risks, including catheter-related infections and higher rates of bile leakage, which might also modulate microbial dynamics (108, 109).

## 9 Antibiotics and the role of microbiota in cancer therapy

Recent studies have shown that the gut microbiota can influence tumor growth remotely. In heterotopic mouse xenograft models, Thomas et al., successfully halted PDAC growth through the administration of broad-spectrum antibiotics (110). This bacterial depletion led to increased expression of tumor suppressor genes such as death-associated protein kinase 2, Krüppel-like factor 9, and Lumican. In contrast, mice with an intact microbiota exhibited upregulation of pro-tumorigenic genes like tenascin C, chemokine ligand 10, and plexin-A4 (110). The immune status of TME in PDAC differed significantly depending on the presence or absence of the gut microbiota. In mice lacking an adaptive immune system (NOD-SCID), an increased number of CD45^+^ innate immune cells infiltrated PDAC tumors when treated with antibiotics. In contrast, untreated mice had fewer CD45^+^ cells, leading to larger tumor size. Moreover, microbial ablation has been shown to significantly slow the progression from pancreatic intraepithelial neoplasia (PanIN) to PDAC *in vivo*. These findings suggest that the intrapancreatic microbiota suppresses immune surveillance and innate immune responses, thus promoting tumor growth (110).

Additionally, in a human study, the depletion of *Klebsiella pneumoniae*, which is associated with gemcitabine resistance, improved patient survival. Bacterial ablation also enhanced the efficacy of checkpoint inhibitors by upregulating programmed PD-1 expression in PDAC mouse models. Interestingly, in renal and non-small cell lung carcinoma bacterial ablation reduced the effectiveness of checkpoint blockade therapy (111), likely due to the broad-spectrum nature of the antibiotics, which may eliminate both pro- and anti-tumorigenic bacterial species (33). This contradiction underscores the importance of selectively targeting harmful bacteria while preserving beneficial species. A more targeted bacterial ablation, combined with systemic therapies, could improve outcomes, especially given that human PDAC tissue predominantly harbors Gram-negative bacteria (34, 61).

## 10 Conclusions

Overall, the findings from these studies emphasize the importance of considering microbiota composition in the treatment and management of PDAC, particularly in patients with biliary stents. This disruption in microbiota composition is associated with activated inflammatory pathways and impaired barrier function, making the host more vulnerable to pathogens. Dysbiosis induced by biliary stenting and chemotherapy not only promotes tumor progression through chronic inflammation and immune modulation but also contributes to chemoresistance, complicating treatment efforts. As highlighted in the review, it would be better to avoid the use of biliary drainage in patients with jaundice due to pancreatic cancer, but clinical practice often differs from theory, so in these cases it is better to prefer transcutaneous biliary drainage rather than endoscopic drainage, used in combination with probiotics.

Probiotics and prebiotics represent promising adjunctive strategies in PC, capable of modulating dysbiosis, enhancing therapeutic response, and reducing treatment-related side effects. These findings support the integration of microbiome-based approaches into future personalized therapies.

Future therapeutic strategies should incorporate approaches to modulate the microbiota, potentially improving treatment efficacy and patient outcomes in PDAC.
